# Risk of acute myocardial infarction after transurethral resection of prostate in elderly

**DOI:** 10.1186/1471-2482-13-S2-S35

**Published:** 2013-10-08

**Authors:** Claudio de Lucia, Grazia Daniela Femminella, Giuseppe Rengo, Antonio Ruffo, Valentina Parisi, Gennaro Pagano, Daniela Liccardo, Alessandro Cannavo, Paola Iacotucci, Klara Komici, Carmela Zincarelli, Carlo Rengo, Pasquale Perrone-Filardi, Dario Leosco, Fabrizio Iacono, Giuseppe Romeo, Bruno Amato, Nicola Ferrara

**Affiliations:** 1 Department of Translational Medical Sciences, Federico II University, Naples, Italy; 2 Salvatore Maugeri Foundation, IRCCS - Scientific Institute of Telese Terme, Benevento, Italy; 3 Department of Urology, Federico II University, Naples, Italy; 4 Department of Advanced Biomedical Sciences, Federico II University, Naples, Italy; 5 Department of General, Geriatric, Oncologic Surgery and Advanced Technologies, Federico II University, Naples, Italy

**Keywords:** Benign prostatic hyperplasia, transurethral resection of prostate, myocardial infarction, elderly, heart failure, physical activity

## Abstract

**Background:**

Benign prostatic hyperplasia is a frequent disease among elderly, and is responsible for considerable disability. Benign prostatic hyperplasia can be clinically significant due to lower urinary tract symptoms that take place because the gland is enlarged and obstructs urine flow. Transurethral resection of the prostate remains the gold standard treatment for patients with moderate or severe symptoms who need active treatment or who either fail or do not want medical therapy. Moreover, perioperative and postoperative surgery complications as cardiovascular ones still occur. The incidence of acute myocardial infarction in patients undergoing transurethral resection of the prostate is controversial. The first studies showed an increase in mortality and relative risk of death from myocardial infarction in transurethral resection of the prostate group vs open prostatectomy but these results are in contrast with more recent data.

**Discussion:**

Given the conflicting evidence of the studies in the literature, in this review we are going to discuss the factors that may influence the risk of myocardial infarction in elderly patients undergoing prostate surgery. We analyzed the possible common factors that lead to the development of myocardial infarction and benign prostatic hyperplasia (cardiovascular and metabolic), the stressor factors related to prostatectomy (surgical and haemodynamic) and the risk factors specific of the elderly population (comorbidity and therapies).

**Summary:**

Although transurethral resection of the prostate is considered at low risk for severe complications, there are several reports indicating that cardiovascular events in elderly patients undergoing this surgical operation are more common than in the general population. Several cardio-metabolic, surgical and aging-related factors may help explain this observation but results in literature are not concord, especially due to the fact that most data derive from retrospective studies in which selection bias cannot be excluded. Subsequently, further studies are necessary to clarify the incidence of acute myocardial infarction in old people.

## Background

Benign prostatic hyperplasia (BPH) is a frequent problem among elderly, and is responsible for significant disability. BPH is a histological diagnosis that refers to the proliferation of smooth muscle and epithelial cells within the prostatic transition zone. The prevalence and incidence of BPH increases with aging. Histologic BPH is present in around 8% of men aged 31 to 40, 50% of men aged 51 to 60, 70% of men aged 61 to 70, and 90% of men aged 81 to 90. Thereby, symptomatic (clinical) BPH is present in approximately 26% of men in the fifth decade of life, 33% of men in the sixth decade, 41% of men in the seventh decade, and 46% of men in the eighth decade of life and beyond. BPH can be clinically relevant due to lower urinary tract symptoms (LUTS) that take place because the gland is enlarged and obstructs urine flow. Urinary symptoms can include: a frequent, urgent need to urinate, difficulty starting urination, slow (prolonged) urination, nicturia, urinary tract infections, hydronephrosis [1]. The enlarged gland seems to contribute to LUTS via at least two mechanisms: 1) direct bladder outlet obstruction from enlarged tissue (static component) and 2) increased smooth muscle tone and resistance within the enlarged gland (dynamic component). Instruments such as the American Urological Association-Symptom Index are now widely utilized to quantify the severity of LUTS in both clinical trials and clinical practice.

The impact of LUTS/BPH on quality of life is very considerable and should not be underestimated [2]. The most important motivations for treatment is symptoms' severity influence on quality life and so the main goal of treatment is to resolve and alleviate storage and voiding symptoms. Hence, treatment has been focused on the amelioration of disease progression and prevention of complications. A variety of pharmacologic classes are used in medical treatment including alpha-adrenergic antagonists (alpha-blockers), 5-alpha-reductase inhibitors, anticholinergics and phytotherapeutics.

The classic surgical interventions include open prostatectomy (OP) and transurethral resection of prostate (TURP). OP is an invasive surgical procedure that is indicated for men whose prostates, in the point of view of the urologist, are too large for TURP for fear of incomplete resection, significant bleeding or the risk of hyponatremia. TURP remains the gold standard treatment for patients with moderate or severe LUTS who need active treatment or who either fail or do not want medical therapy. TURP was developed during the 1930s as a less invasive alternative to OP in the treatment of benign prostatic enlargement [3]. With increasing worldwide attention on health care costs, minimally invasive therapies for the management of BPH (as Transurethral needle ablation, Transurethral microwave therapy - TUMT, Transurethral incision of the prostate, holmium laser enucleation of the prostate, laser photovaporization of the prostate) will become progressively more important in cost-effectiveness evaluations.

However, in the absence of strong evidence favouring newer technologies, TURP currently remains clinically and cost effective. Moreover, TURP peri-operative and post-operative complications still occur. Post-operative complications of TURP are retrograde ejaculation (60-90% after TURP), urinary tract infections (cause by bacterial colonization of the prostate; occurs in 2%), persistent urinary retention (2.5% went home from the hospital with catheter), bladder neck stricture (occurs 2-10%), urethral stricture (around 10%; limiting the time of the transurethral catheter), cardiac complications (acute myocardial infarction - AMI). The incidence of AMI in patients undergoing TURP is controversial. The first long-term study by Roos et al. comprised 54.000 patients from Canada, England and Denmark and showed an increase in mortality of 40 % after a follow-up period of 2-7 years. The relative risk of death from AMI was 2.5 in the TURP group. In addition in this study, TURP was less effective than OP [4].

However some confounding factors could not be ruled since the two populations are not comparable due to the tendency to assign older and less healthy men to TURP because it is less traumatic. Furthermore those studies designed retrospectively did not distinguish possible prognostic factors which were partially responsible for the assignment of the patients to TURP or OP. On the other side, in a study by Shalev at al. there were no differences in the long-term incidence of AMI after open or transurethral prostatectomy. Indeed, AMI developed in the first 3 years after operation in 6.3% TURP patients compared to 6.9% OP patients. Neverthless, in men with BPH after both surgical procedures the incidence of long-term AMI seems to be higher than in the general male population of the same age group [5]. Hahn et al. reported a long-term incidence of AMI of 7.9% after TURP and of 5.2% after TUMT. As the incidence of AMI in both study arms was higher than in the general population (standard morbidity ratio: 1.5), the authors proposed that BPH rather than the treatment itself is associated with cardiovascular disease. The similarity of the results for TURP and TUMT prompts that the prostatic enlargement rather than the treatment is associated with cardiovascular disease [6]. Another study also confirmed a similar mortality or incidences of AMI in TURP group compared to OP at all timepoints (1, 5 and 8 years). Is important to remember the retrospective nature of this analysis and that although age of both groups was identical, the comorbidity rate was slightly (25.2% vs. 21.2%) higher in the TURP-group [7]. AMI remains the principal cause of hospitalizations as well as the leading cause of death worldwide. In patients with AMI who are older than 70 years, mortality rates exceed 30% [8]. The incidence and prevalence of MI increase progressively with age. In the United States, over 60% of AMI occur in patients 65 years of age or older, and approximately one third occur in persons over age 75 [9].

Coronary heart disease is more advanced in the elderly than in younger patients. Three-vessel disease occurred in 44% of subjects with ischemic heart disease aged 65-74 and in 63% of subjects aged 75 and over [10,11].

Heart failure is common in the elderly population. Approximately 6 to 10 percent of the population 65 years or older have heart failure. In patients older than 67 years, median survival is generally less than 3 years after hospitalization for heart failure [12,13]. Heart failure is the most common reason for hospitalization in elderly patients. Etiology of heart failure is often multifactorial in the elderly [14-16]. The common causes of heart failure include ischemic heart disease, valvular heart disease, hypertensive heart disease, and cardiomyopathy. The complexity in the presentation and in the evolution of heart failure in the elderly leads to research new treatments that can improve the survival of patients with this debilitating disease [17,18].

Given the conflicting results of the studies in the literature on AMI incidence in patients undergoing TURP or OP, in this review we are going to discuss the factors that may influence the risk of AMI in elderly patients undergoing prostate surgery. We therefore decided to analyze the possible common factors that lead to the development of AMI and BPH (cardiovascular and metabolic), the stressor factors related to prostatectomy (surgical and haemodynamic risk factors) and the risk factors specific of the elderly population (comorbidity and therapies).

## Discussion

### Cardiovascular and metabolic factors

Some evidence indicates that the same risk factors associated with heart disease may increase the risk of developing BPH. These risk factors include obesity, diabetes, diet, dyslipidemia, hypertension [19].

Obesity strikingly increases the risk of BPH. The risk of BPH increases by 10% for each 0.05-increase in the waist-to-hip ratio. Increased adiposity is positively related with radiographically determined prostate volume and enlargement, suggesting that obesity promotes prostate growth and rises the risk of clinical BPH and LUTS [20]. Abdominal obesity increases the estrogen-to-androgen ratio and may lead sympathetic overactivity, both hypothesized to influence the development of BPH and the severity of urinary obstructive symptoms. The increase in adipose aromatization of testosterone to estrogen and the altered levels of testosterone and sex hormone binding globulin in patients with raised adipose stores promote prostatic epithelial and stromal proliferation. Several mechanisms have been proposed to explain the relationship between obesity and increased sympathetic tone. Inflammation with elevated serum concentrations of IL-6 and TNF-a, increased insulin levels in proportion to the amount of adipose stores and leptin increase have been shown to result in sympathetic overdrive. Furthermore activation of the renin- angiotensin-aldosterone system and adrenal alfa2-adrenergic downregulation and desentitation may enhance sympathetic tone [21-25].

Diabetes may also significantly influence the risk of BPH and LUTS in older men. During insulin-resistance, hyperinsulinemia develops to conuteract the decreased responsiveness of the body to insulin. Although the compensatory hyperinsulinemia prevents development of fasting hyperglycemia in insulin-resistant individuals, the increased level of circulating insulin directly and/or indirectly affects different molecular signaling and can promote prostatic growth. Hyperinsulinemia stimulates the liver to produce more insulin-like growth factor (IGF), another mitogen and an anti-apoptotic agent which binds insulin receptor/IGF receptor and stimulates prostate growth. The levels of IGFs and IGF binding proteins in prostate tissue and in blood are associated with BPH risk [26,27]. It has been demonstrated that a healthy lifestyle plays a critical role in cardiovascular health. It is now apparent that the same is true in the development of BPH. Men with high energy intakes and high intake of protein and polyunsaturated fatty acid were at greater risk of developing BPH [28]. Daily aerobic exercise can ameliorate methabolism, particularly when combined with a low-fat, high-fiber diet consisting of whole grains, fruits, and vegetables. In cell culture studies, this type of lifestyle regimen has recently been shown to reduce the growth of serum-stimulated prostate epithelial cells and the growth of androgen-dependent prostate cancer cell lines. Alcohol consumption is associated with a decreased likelihood of BPH but not of LUTS. The association is thought to be related to alcohol's cardiovascular effects and modulation of steroid hormone metabolism [29]. The prostate synthesizes and stores large amounts of cholesterol and prostate tissues may be particularly sensitive to modifications in cholesterol metabolism. Hypercholesterolemia, a major risk factor for cardiovascular disease, is also a risk factor for BPH. Experimental and clinical findings indicate that agents that inhibit cholesterol absorption from the intestine can reduce prostate gland size and improve LUTS [30].

A considerable, age independent association exists between BPH symptoms and hypertension. This finding indicates in increased sympathetic activity a common pathophysiological factor for both disease states [31]. Elderly patients with nicturia had a higher blood pressure and higher serum catecholamine levels compared to healthy elderly controls. Furthermore, hypertension itself seems to have an adverse influence on LUTS and currently used alfa1-adrenergic receptor antagonists (all except tamsulosin), were originally developed as antihypertensive drugs.

### Surgical risk factors

An important stressful factor to the heart during TURP procedure is the fluid excess due to absorption of fluid that enters the circulation via blood vessels in the resection area. Small amounts are probably of little consequence, but the accidental transfer of > 3 L of fluid results in symptoms originating from the cardiovascular system as well as bradycardia and hypotension and sometimes involving the central nervous system with neurologic disturbance as confusion, aggressiveness and depressed consciousness.

The fully developed 'TURP syndrome' is a very rare systemic complication, caused by excessive absorption of electrolyte-free irrigation fluids but mild forms are quite common. The incidence of 'TURP syndrome' has decreased significantly during the last few decades from 3% to <1% but this complication is very dangerous because characterised by symptoms changing from an asymptomatic hyponatremic state to convulsions, coma and death.

Symptoms may occur if the amount of fluid absorbed exceeds 1 litre, which occurs in between 5 and 10% of the TURPs performed [32]. Normal saline cannot be used as irrigation solution with conventional monopolar resection. Glycine solution is generally used as an irrigation solution in traditional therapeutic endoscopic urologic procedures [33].

The role of irrigation solution is to distend the bladder, clear the surgical site and wash away residual tissue and blood. Various irrigation fluids (glycine, sorbitol, mannitol and normal saline) have been used for TURP. Glycine solution is the most commonly used irrigant in traditional therapeutic endoscopic urologic procedures.

To measure serum electrolytes (sodium and potassium levels) changes during TURP surgery is a simple and economical method for indirect assessment of fluid absorption for early identification of TURP syndrome [34]. Several reports showed that glycine absorption causes neurologic symptoms and echocardiogram changes, as well as T-wave depression or inversion on electrocardiography for up to 24 hours after surgery. Other ECG alterations due to 1.5 glycine absorption are prolongation of the RR interval and QRS duration.

Another significant surgical risk factor during TURP is bleeding and erythrocyte transfusions may subsequently be necessary. Considerable perioperative bleeding will impair the visibility of the surgical field, reduce the tissue elimination and raise hypovolemia risk. Hemorrhages are one of the major intraoperative complications with 1 litre or more blood loss in 14% of patients [35,36].

Transfusion rate decreased in last ten years from 22% to 0.4-7.1%. Technological improvements of monopolar and bipolar TURP, microprocessor-controlled units and better instrumentarium resulted in a significant decrease of transfusion rates. Some authors have emphasized the decreased amounts of intra- and postoperative bleeding when the bipolar device is used. The bipolar electrosurgical equipment simultaneously vaporizes the tissue and controls the bleeding. Because TURP syndrome and blood loss are strictly related, Huang et al., showed that bipolar TURP resulted in deeper coagulation zones, less bleeding, less intraoperative hemoglobin drop, smaller serum sodium decrease, and therefore no TURP syndrome occurred [37]. Important bleeding during surgery can be also almost eliminated with alternative energy sources such as the Holmium laser or Potassium Titanyl Phosphate laser.

### Hemodynamic factors

Besides the above mentioned data on TURP and its correlation with AMI incidence, hemodynamic factors have been suggested as potential triggers of myocardial ischemia during TURP. In particular, it has been reported that body temperature lowering due to cold irrigating fluid may result in "cardiac stress" as demonstrated by increased peripheral resistance and arterial pressure, as well as reduced stroke volume and cardiac output after TURP compared to control procedures [38]. Other studies, however, have demonstrated that patients subjected to OP and TURP presented the same degree of hemodynamic alterations, without significant differences, implying that the kind of surgery does not play a relevant role in the late mortality rate of these patients [39]. Further evidence on the hypothesis of TURP-related cardiac stress come from studies evaluating cardiac enzyme levels after this surgical procedure. A slight increases in creatinine kinase-B subunit and lactate dehydrogenase 1 has been reported in patients undergoing TURP, but the criteria for AMI were never fulfilled [40]. More recently, it has been shown that Troponin T is not increased in patients undergoing TURP without previous cardiac problems. Pro-brain natriuretic peptide is modestly increased but it does not indicate significant ventricular dysfunction [41,42].

Thus, the clinical significance of these increases remains unknown and it is likely that patients with a history of cardiac disease are at a higher risk of myocardial damage during TURP. On the other hand, in elderly patients with no history of heart disease undergoing TURP, continuous ECG Holter monitoring during the pre- intra- and post-operative periods was not able to detect significant variations in electrocardiographical signs indicative for ischemic disease [43].

### Comorbidities and therapies

It is well established that benign prostatic hyperplasia is associated with old age and both OP and TURP are performed in patients with multiple comorbidities. The study by Roos et al. was the first to compare long-term mortality after TURP to OP, showing that TURP resulted in higher long-term mortality compared to OP [4]. However, this study, as mentioned above, was retrospective and confounding factors leading to altered patients' selection could not be ruled. In fact, given the less invasive nature of TURP compared to OP, it is likely that patients with multiple comorbidities and higher surgical risk are preferentially referred to TURP, leading to a selection bias that cannot be eliminated in retrospective studies. Moreover, although some studies have confirmed a direct effect of comorbidity on perioperative morbidity and mortality after these procedures [44-46].

Recent evidence indicate that both interventions result in similar cardiovascular outcome. Madersbacher et al. have shown that despite the fact that comorbidity rate was slightly higher in patients undergoing TURP compared to OP, no excess risk of AMI or death after TURP were observed [7]. However, in studies comparing TURP to other mini-invasive techniques, such as TUMT, it has been shown that both interventions are followed by a higher incidence of AMI compared to the general population in patients suffering from various comorbidities (asthma, cardiovascular disease, diabetes) [6]. A recent study specifically evaluated the effect of comorbidities on outcome after TURP and OP indicating that, despite the high prevalence of comorbid conditions in this population, no comorbidity taken alone can be considered an absolute contraindication for these procedures and is associated with the occurrence of complications [47].

Another specific feature of the elderly population is the polypharmacotherapy, that is strictly related to increased comorbidity. Anticoagulant and antiplatelet drugs are widely prescribed in elderly patients, especially those with increased cardiovascular risk. Some studies have evaluated how the management of these drugs can affect outcome in patients undergoing TURP, since stopping anticoagulants prior to TURP may reduce peri-operative blood loss, but it may also increase the risk of cardiovascular events. A recent survey conducted in the United Kingdom has shown that there is a wide variation in stopping aspirin before TURP among urologists [48]. In particular, aspirin is stopped also in patients with increased cardiovascular risk factors, exposing them to the risk of complications. However, a retrospective analysis has indicated that men who have anticoagulation therapy stopped before TURP do not have a higher incidence of cardiovascular, cerebrovascular or thromboembolic events, or bleedings when compared with anticoagulant-naïve patients [49,50]. It has also been reported that in patients undergoing TURP a hypercoagulable state can be detected and that surgery itself increases the risk of thrombogenic events [51], thus it is not safe to leave high-risk patients without effective anticoagulation, but to address this issue further studies are needed.

As for lifestyle habits and non-pharmacological interventions, some studies have shown that physical activity may have a protective role on prostatic hyperplasia [52-54]. In particular, it has been observed that an inverse relationship exists between physical activity and prostatic hyperplasia with a risk reduction for both incident surgery and symptomatic cases among non-sedentary men [55-58]. It has been hypothesized that reduction in sympathetic nervous system activity resulting from regular physical training is also able to reduce prostatic smooth muscle tone. In fact, it is well established that aging is associated with altered sympathetic tone and that exercise training has beneficial effects on cardiovascular risk profile in the elderly.

## Summary

Although urological surgery - and TURP in particular - is considered at low risk for subsequent severe complications, there are several reports indicating that cardiovascular events in elderly patients undergoing these procedures are more common than in the general population. Several cardio-metabolic, surgical and aging-related factors may help explain this observation but the picture still remains confusing, especially due to the fact that most data derive from retrospective studies in which selection bias cannot be excluded. Moreover, recent guidelines suggest to consider patients aged more than 60 years with no history of cardiopulmonary disease at intermediate surgical risk, indicating that age per se plays a role in surgery-specific risk [59].

Thus, the efforts to reduce cardiovascular complications in elderly patients should continue, focusing on better stratification of the risk of atherosclerotic coronary artery disease, detection of the presence of ischemia, and management of surgery-related cardiovascular stressors. In this vein, prospective studies might help clarify unsolved clinical issues and identify pathophysiological mechanisms underlying increased cardiovascular risk in urological elderly patients.

## List of abbreviation used

BPH: benign prostatic hyperplasia; TURP: transurethral resection of prostate; LUTS: lower urinary tract symptoms; OP: open prostatectomy; AMI: acute myocardial infarction; TUMT: transurethral microwave therapy.

## Competing interests

The authors declare that they have no competing interests.

## Authors' contributions

CdL and GDF: conception and design, final approval of the version to be published; GR, AR,VP,GP, DL, AC, PI, KK, GR: drafting the manuscript, final approval of the version to be published; PPF, DL, FI, NF: revising the manuscript for important intellectual content, final approval of the version to be published.

## Declarations

Publication costs for this article were funded by the Dipartmento di Scienze Mediche Traslazionali, Naples, Italy.

This article has been published as part of *BMC Surgery *Volume 13 Supplement 2, 2013: Proceedings from the 26th National Congress of the Italian Society of Geriatric Surgery. The full contents of the supplement are available online at http://www.biomedcentral.com/bmcsurg/supplements/13/S2
